# Rice *Brittle Culm19* Encoding Cellulose Synthase Subunit CESA4 Causes Dominant Brittle Phenotype But has No Distinct Influence on Growth and Grain Yield

**DOI:** 10.1186/s12284-021-00536-2

**Published:** 2021-11-25

**Authors:** Xiaozhi Ma, Chunmei Li, Rui Huang, Kuan Zhang, Qian Wang, Chongyun Fu, Wuge Liu, Changhui Sun, Pingrong Wang, Feng Wang, Xiaojian Deng

**Affiliations:** 1 State Key Laboratory of Crop Gene Exploration and Utilization in Southwest China, Rice Research Institute, Sichuan Agricultural University, Chengdu, 611130 China; 2 Guangdong Provincial Key Laboratory of New Technology in Rice Breeding, Rice Research Institute, Guangdong Academy of Agricultural Sciences, Guangzhou, 510642 China; 3 Guangzhou Key Laboratory for Research and Development of Crop Germplasm Resources, College of Agriculture and Biology, Zhongkai University of Agriculture and Engineering, Guangzhou, 510225 China; 4 Center for Agricultural Genetic Resources Research, Shanxi Agricultural University, Taiyuan, 030031 China

**Keywords:** Rice, Dominant mutation, Brittle culm, Cellulose synthase, Secondary cell wall

## Abstract

**Background:**

Mechanical strength is a crucial agronomic trait in rice (*Oryza sativa*), and brittle mutants are thought suitable materials to investigate the mechanism of cell wall formation. So far, almost all brittle mutants are recessive, and most of them are defected in multiple morphologies and/or grain yield, limiting their application in hybrid breeding and in rice straw recycling.

**Results:**

We identified a semi-dominant brittle mutant *Brittle culm19* (*Bc19*) isolated from the *japonica* variety Nipponbare through chemical mutagenesis. The mutant showed the same apparent morphologies and grain yield to the wild type plant except for its weak mechanical strength. Its development of secondary cell wall in sclerenchyma cells was affected, along with reduced contents of cellulose, hemicellulose, lignin and sugars in culms and leaves. Positional cloning suggested that the *Bc19* gene was allelic to *OsCESA4*, encoding one of the cellulose synthase A (CESA) catalytic subunits. In this mutant, a C-to-T substitution occurred in the coding sequence of *BC19*, causing the P507S missense mutation in its encoded product, which was located in the second cytoplasmic region of the OsCESA4 protein. Furthermore, introducing mutant gene *Bc19* into the wild-type plant resulted in brittle plants, confirming that the P507S point mutation in OsCESA4 protein was responsible for the semi-dominant brittle phenotype of *Bc19* mutant. Reverse correlation was revealed between cellulose contents and expression levels of mutant gene *Bc19* among the homozygous mutant, the hybrid F_1_ plant, and the *Bc19* overexpression transgenic plants, implying that gene *Bc19* might affect cellulose synthesis in a dosage-dependent manner.

**Conclusions:**

*Bc19*, a semi-dominant brittle mutant allele of gene *OsCESA4*, was identified using map-based cloning approach. The mutated protein of *Bc19* possessing the P507S missense mutation behaved in a dosage-dependent semi-dominant manner. Unique brittle effect on phenotype and semi-dominant genetic quality of gene *Bc19* indicated its potential application in grain-straw dual-purpose hybrid rice breeding.

**Supplementary Information:**

The online version contains supplementary material available at 10.1186/s12284-021-00536-2.

## Background

Mechanical strength is one of the most important traits for cereal crops, and is determined by plant cell walls, which constitute the skeletal structures of the plant bodies. Ninety-percent components of plant cell walls are polysaccharides, which exist mainly as cellulose and hemicellulose in primary cell walls (PCW) and secondary cell walls (SCW), and as lignin only in SCW (Darley et al. 2001; Taylor et al. 2000). SCW generally determines the mechanical strength of cell walls, so defects in biosynthesis of cellulose, hemicellulose and/or lignin always result in inferior mechanical index and brittle plant bodies, which in turn make these brittle culm mutants valuable materials for understanding the mechanism of SCW formation.

Till now, a number of brittle mutants have been studied, and some responsible genes have been identified in *Arabidopsis thaliana*, rice (*Oryza sativa*) and other cereal crops, which to some extent revealed the mechanism regulating mechanical strength of the plant body and metabolic pathway of plant cell walls. Initially, the physical characteristics of crop stems were first described through three barley brittle mutants, in which the brittle phenotype was due to fewer numbers of cellulose molecules in the cell walls (Kokubo et al. 1989, 1991). Later on, three Arabidopsis *irregular xylem* mutants, *irx1*, *irx3* and *irx5*, were identified (Turner and Somerville 1997), and the relevant corresponding genes were found to encode the three basic cellulose synthase A (CESA) catalytic subunits, AtCESA8, AtCESA7 and AtCESA4, respectively (Taylor et al. 1999, 2000, 2003). In addition, several genes involved in other different steps of cell wall formation were reported in Arabidopsis. *IRX4* encoding a cinnamoyl-CoA reductase (CCR) is essential for lignin biosynthesis; *FRAGILE FIBER1* (*FRA1*) encodes a kinesin-like protein and regulates the oriented deposition of cellulose microfibrils; and mutant of gene *FRA2* is attributable to altered fiber cell elongation and expansion (Jones et al. 2001; Zhong et al. 2002; Burk et al. 2001).

Coincidentally, identification of some brittle mutants in rice caused by *Tos17* also revealed three catalytic subunits essential for cellulose synthesis, OsCESA4, OsCESA7, and OsCESA9, which are homologous to AtCESA8, AtCESA4 and AtCESA7, respectively (Tanaka et al. 2003). Up to now, at least 25 mutants exhibiting brittle leaves and/or culms have been reported in rice, some of which turned out to be different mutant alleles of *CESAs*, such as *bc7*, *bc11*, *Bc6* and *fc17* (Yan et al. 2007; Zhang et al. 2009; Kotake et al. 2011; Li et al. 2018). In total, 12 genes responsible for brittle traits are cloned, which directly or indirectly participate in cellulose biosynthesis and cell wall formation (Kotake et al. 2011; Wu et al. 2012; Vega-Sánchez et al. 2012; Wang et al. 2016b). *Brittle Culm1* (*BC1*) encoding a COBRA-like protein regulates cellulose assembly by modulating cellulose crystallite size (Li et al. 2003; Liu et al. 2013). *BC3* is essential for proper SCW construction, and its encoding protein functions in membrane dynamics (Hirano et al. 2010). A dual-targeting kinesin protein encoded by *BC12* is involved in cell-cycle progression and cellulose microfibril deposition (Zhang et al. 2010). Proteins encoded by *BC10*, *BC14* and *BC15* are all localized in the Golgi complex, but they function differently in regulating cellulose synthesis (Zhou et al. 2009; Zhang et al. 2016, 2011; Song et al. 2011; Wu et al. 2012). These studies have uncovered some important biochemical processes in cell wall formation and remodeling, while our understanding of cell wall biosynthesis and modification is still limited.

Almost all brittle mutants are recessive, such as *bc1*, *bc3*, *bc11* and *fc17*, except *Bc6*, a semi-dominant mutant of *OsCESA9* (Li et al. 2003, 2018; Hirano et al. 2010; Zhang et al. 2009; Kotake et al. 2011). Meanwhile, most brittle plant bodies were also aberrant in multiple morphologies, interfering with their application in rice breeding. For example, decreased plant height and shorter roots were often seen in these mutants, such as *bc3*, *bc10*, *bc11* and *bc12* (Hirano et al. 2010; Zhou et al. 2009; Zhang et al. 2009, 2010). Fertility and/or tillering were severely affected in some mutants, including NE1031, *S1*-*24*, *S1*-*60* and *bc10* (Tanaka et al. 2003; Wang et al. 2016a, 2012; Zhou et al. 2009). In the present study, we isolated a semi-dominant brittle mutant *Brittle culm19* (*Bc19*) through chemical mutagenesis. Cellulose, hemicellulose and lignin contents were all reduced in culms and leaves of *Bc19*, and its SCW was much thinner than the wild type plant, while neither apparent morphologies nor grain yield was altered in the mutant. Through map-based cloning, we confirmed that the mutant gene *Bc19* was allelic to *OsCESA4*, and the resulting P507S point mutation was responsible for the brittle phenotype. We suggest that the semi-dominant mutant gene *Bc19* could be a potential genetic resource for implementing high grain yield, heavy biomass and their efficient utilization in breeding grain-straw dual-purpose hybrid rice.

## Results

### Characterization of the *Bc19* Mutant

**Fig. 1:**
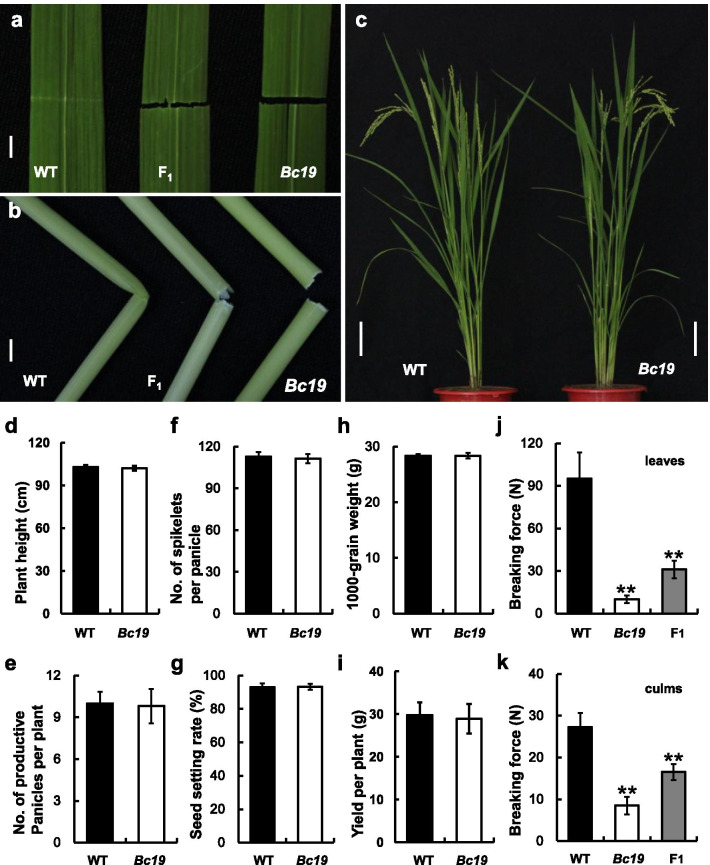
Comparison of phenotypes (**a**–**c**), major agronomic traits (**d**–**i**) and breaking force (**j** leaves and **k** culms). **a** Leaf. **b** The second internode of main culm from the top. **c** Plants two weeks after heading. Bars represent standard deviations of three independent measurements. WT: Nipponbare; F_1_: Obtained from mutant *Bc19* crossing with WT. Double-asterisk signifies statistically significant difference compared to the wild type at *p* < 0.01. Scale bar equals 1 cm in **a**–**b** and 10 cm in **c**

*Bc19* was a brittle mutant obtained from the *japonica* rice cv. Nipponbare by chemical mutagenesis. Its leaves and internodes could be easily broken, showing smooth breakpoints (Fig. 1a, b). The brittle phenotype was milder at the seedling stage and became more severe, especially after heading. Not only culms and leaves, but the weak mechanical strength also went through the whole plant body of *Bc19*, including leaf sheaths, panicle branches, glumes and roots. Strengths for breaking leaves and culms were reduced by approximately 90% and 70% in *Bc19*, respectively (Fig. 1j, k). However, different from most brittle mutants reported, apparent morphology and major agronomic traits were not affected in the *Bc19* mutant, exhibiting comparable plant height, the number of productive panicles per plant and spikelets per panicle, seed setting rate, 1000-grain weight, and grain yield to the wild type plant (Fig. 1c–i).

**Fig. 2:**
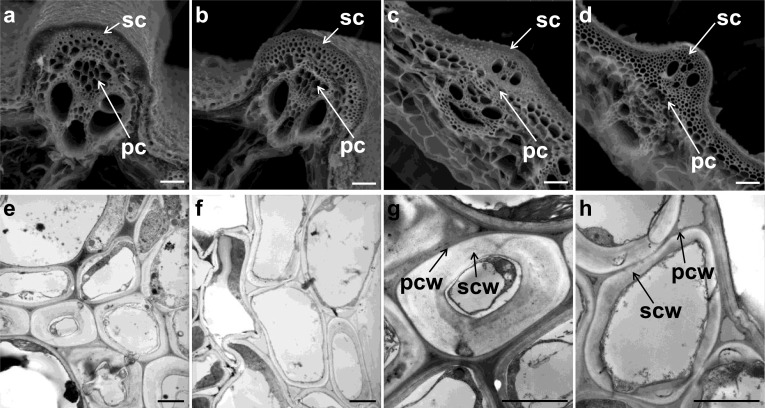
Environmental scanning electron microscope images (**a**-**d**) and transmission electron microscope images (**e**–**f**) of *Bc19* mutant and wild-type plant. **a** and **b** Transverse section of the wild-type and *Bc19* mutant leaf vein, respectively. **c** and **d** Transverse section of the wild-type and *Bc19* mutant culm, respectively. **e** and **f** Sclerenchyma cells of the wild type and *Bc19* mutant, respectively. **g** and **h** Single cell in sclerenchyma tissues of the wild type and *Bc19* mutant, respectively. sc, sclerenchyma tissues; pc, parenchymatous tissues; pcw, primary cell wall; scw, secondary cell wall. Scale bar equals 40 μm in **a**–**d** and 10 μm in **e**–**h**

Decreased mechanical strength in the *Bc19* mutant may result from irregular cell arrangement, cell wall structure and thickness. Therefore, we examined cross sections of leaves and internodes of the wild-type plant and *Bc19* mutant using an environmental scanning electron microscope and a transmission electron microscope (Fig. 2). Obviously, cell walls of sclerenchyma tissues were much thinner in *Bc19*, in both leaf veins and internodes, while cell walls of parenchyma tissues showed no obvious differences (Fig. 2a–d). Moreover, SCW of *Bc19* was found to be thinner, and the layered structure of SCW was not distinct compared with the wild-type plant (Fig. 2e–h). These results suggested that the mutation traits of *Bc19* were very likely associated with a decrease in cell wall thickness, especially the SCW in sclerenchyma tissues which provided the basal structural support of the plant body.

### Alterations in Cell Wall Composition

**Fig. 3:**
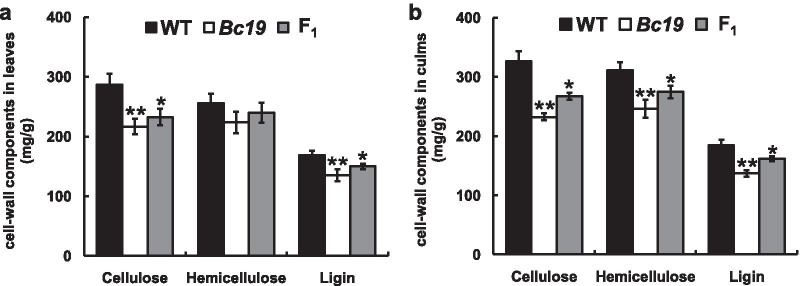
Contents of cellulose, hemicellulose and lignin in cell walls from leaves (**a**) and culms (**b**) of *Bc19*, the wild type (WT) and F_1_ plants, in mg g fresh weight^−1^. F_1_: Obtained from *Bc19* crossing with WT. Double and single Asterisks indicate statistically significant differences compared to the wild type at *p* < 0.01 and *p* < 0.05, respectively

As inferior cell wall formation may result from altered cell wall compositions, we compared cellulose, hemicellulose, and lignin contents between the *Bc19* mutant and the wild-type plant. As shown in Fig. 3, all of the amounts were reduced in leaves and culms of the *Bc19* mutant. Cellulose reductions of 24.1% in leaves and 28.5% in culms were observed in *Bc19*, and lignin contents decreased 20.1% in leaves and 25.9% in culms compared to those in the wild type (Fig. 3). A relatively slight decrease of hemicellulose content occurred in the mutant, 12.5% in leaves and 20.9% in culms (Fig. 3). Subsequently we performed a quantitative analysis of monosaccharides in *Bc19* and wild type plant culms through HPLC assay. As expected, glucose, which makes up the cellulose, and xylose, the main component of hemicellulose, were all decreased in the *Bc19* mutant (Table 1). These results indicated that mutation in gene *Bc19* affected cell wall biosynthesis through alterations of these major components.

**Table 1:** Comparison of monosaccharides composition between the wild type (WT) and *Bc19*

Sugar	WT	*Bc19*	Compared with WT (%)
Glucose	9653 ± 617	7963 ± 75	− 17.5*
Fructose	8568 ± 455	8135 ± 259	− 5.1
Xylose	321 ± 38	251 ± 24	− 21.8*
Arabinose	8.7 ± 0.6	8.4 ± 1.4	− 3.4
Galactose	11.3 ± 0.9	12.2 ± 1.1	8.0
Rhamnose	119 ± 11	123 ± 3	3.4
Mannose	48.1 ± 4.2	61.0 ± 2.4	26.8**

The data are given as means ± SE of three independent repeats. Each monosaccharide was formulated as µg g fresh weight^−1^. Single and double asterisks signify statistical differences compared to the wild type at *p* < 0.05 and *p* < 0.01, respectively

### Map-Based Cloning of the *Bc19* Mutant Gene

The *Bc19* mutant was crossed with three rice cultivars, including the wild-type Nipponbare, and two *indica* cultivars, Minghui 63 and G46B. All of the F_1_ plants from the three crossings tended to be brittle. Meanwhile, milder phenotypes of weak mechanical strength and decrease in breaking force were observed in F_1_ plants producing from crossing between *Bc19* and the wild type (Fig. 1a, b, j, k). Correspondingly, the reduction of cell wall components in F_1_ was also milder than those in the homozygous mutant *Bc19* (such as 18.9% and 18.1% of cellulose decrease in the F_1_ leaves and culms, respectively) (Fig. 3). Therefore, the mutant gene might act in a semi-dominant manner. Furthermore, three quarters of F_2_ plants derived from the three crosses between *Bc19* and normal cultivars were also brittle plants (Additional file 1: Table S1). Therefore, the *Bc19* mutant phenotype should be controlled by a semi-dominant nuclear gene.

**Fig. 4:**
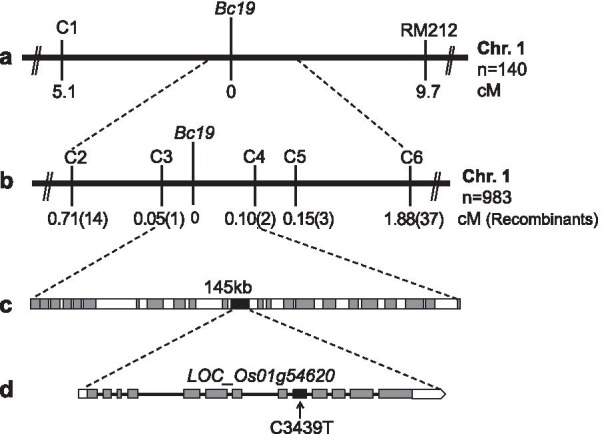
Molecular mapping of *Bc19* locus. **a** The *Bc19* locus was mapped between InDel marker C1 and SSR marker RM212 on the long arm of chromosome 1 (Chr.1) of rice using 140 recessive F_2_ plants. **b** *Bc19* was narrowed between InDel markers C3 and C4 depending on analysis of 983 recessive F_2_ plants. **c** 23 annotated genes were found in the 145-kb region between markers C3 and C4. **d** Structure of the candidate gene *Bc19* (*LOC*_*Os01g54620*). Gray boxes indicate exons and lines indicate introns. The C3439T point mutation is marked with a black arrow

To fine map the *Bc19* locus, an F_2_ population of up to 4000 plants from a cross between *Bc19* and Minghui 63 was generated. Initially, we analyzed 140 normal plants from this F_2_ population with more than 300 SSR markers and located the *Bc19* gene on the long arm of rice chromosome 1, with 9.7 cM to RM212 (Fig. 4a). Then several InDel markers on both sides of RM212 were developed based on sequence polymorphism between *japonica* cv. Nipponbare and *indica* cv. 93–11, and the *Bc19* gene was mapped between RM212 and InDel marker C1, with 5.1 cM to C1 (Fig. 4a, Additional file 1: Table S2). To narrow the *Bc19* locus, we designed more InDel markers within this region and analyzed 983 normal plants in total. Finally, we narrowed the *Bc19* locus down to a 145-kb interval between InDel markers C3 and C4, with the genetic distance of 0.05 cM and 0.10 cM, respectively (Fig. 4b, c, Additional file 1: Table S2).

According to the gene annotation information provided by the MSU Rice Genome Annotation Release 7 (http://rice.plantbiology.msu.edu), this region contains 23 genes (Fig. 4c), of which 8 are transposons. Based on the putative functions of the rest 15 genes, *LOC*_*Os01g54620* encoding OsCESA4 is most likely the candidate gene, which was previously identified essential for cellulose synthesis (Tanaka et al. 2003). Therefore, we sequenced and compared this gene from the genomic DNA of *Bc19* and wild-type plants, finding one base-pair C-to-T substitution at position 3439 (Fig. 4d). Through reverse transcription (RT)-PCR, the corresponding C-to-T substitution at position 1519 of the gene's cDNA sequence was revealed, turning CCA into TCA and resulting in the 507th amino acid residue, proline, replaced with serine.

Sequence analysis suggested that *BC19* gene contained 13 exons and 12 introns with 5743-bp genomic DNA and 2970-bp cDNA. Multiple alignments indicated that the missense mutation from proline to serine at the 507th residue of Bc19 protein is strictly conserved among CESA family members in rice, Arabidopsis and several other species, which may affect the proper function of OsCESA4 (Additional file 2: Fig. S1 and S2). Nonetheless, the predicted three dimension structure of Bc19 was not affected by its P507S mutation, which is located between two β-pleated sheets (Additional file 2: Fig. S3).

### Functional Identification of *Bc19*

**Fig. 5:**
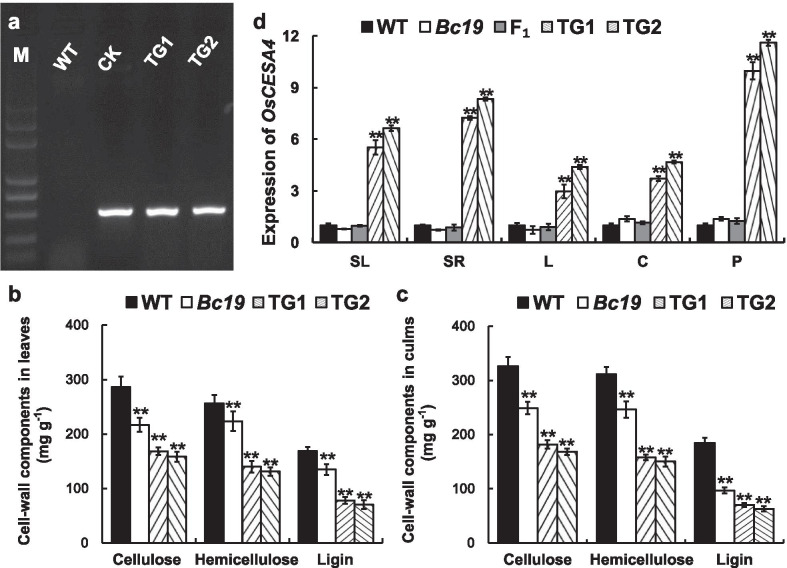
Functional identification of *Bc19*. **a** PCR assay of transgenic lines of brittle gene *Bc19*. M, DL-2000 plus marker; WT, the wild-type parent Nipponbare (the transgenic acceptor, as PCR-negative control); CK, *pC2300-Bc19* plasmid (PCR-positive control); TG1 and TG2, positive transgenic lines. **b** and **c** Comparison of cell wall components among the wild type plant, mutant *Bc19* and two positive transgenic lines, in leaves and culms, respectively. **d** Transcript levels of *OsCESA4* in different tissues of the wild type (WT), the mutant *Bc19*, the hybrid F_1_ plants from a cross between WT and *Bc19*, and two transgenic lines (TG1 and TG2), respectively. SL, seedling leaves; SR, seedling roots; L and C, leaves and culms from plants two weeks after heading, respectively; P, young spikelets. The expression data of WT were all set to 1.0 and those of other plants were adjusted accordingly. Bars represent standard deviations of three independent experiments. Double asterisks signify statistically significant differences compared to WT at *p* < 0.01

A transgenic experiment was conducted to confirm that mutation in gene *LOC*_*Os01g54620* is responsible for the brittle phenotype of *Bc19*. The plasmid *pC2300*-*Bc19* containing the coding region of the mutant gene *Bc19* right after the rice *Actin1* promoter was introduced into the wild-type plant. As a result, 13 independent transgenic plants were obtained, of which 11 plants were identified as positive transgenic plants by PCR with a pair of primers extending from the plasmid to the coding sequence of *Bc19* (Fig. 5a, showing 2 among 11 lines). As expected, independent transgenic lines carrying the mutant gene *Bc19* showed obviously brittle phenotype. Moreover, the decrease in cell wall components of transgenic lines TG1 and TG2 were severer than those of mutant *Bc19* (Fig. 5b, c), probably because of their higher transcript levels of mutant gene *Bc19* guided by the strong promoter of *Actin1* (Fig. 5d). These results suggested that the brittle phenotype of the *Bc19* mutant was due to the P507S substitution of OsCESA4, and the mutant *Bc19* gene blocked the synthesis of essential components of cell wall in a dominant way.

### qRT-PCR Analysis of *BC19* and the Related Genes

To analyze the function of *BC19* gene and how the mutant gene impacted its cell wall synthesis, expression patterns of *BC19* and other 5 related genes were assessed in both wild-type plants and the *Bc19* mutant by qRT-PCR. *OsCESA4*, *OsCESA7* and *OsCESA9*, encoding three catalytic subunits of CESA, are essential for β-1,4-glucan synthesis and are not redundant during SCW formation (Tanaka et al. 2003; Doblin et al. 2002). *BC1* encodes a COBRA-like protein and participates in SCW formation, and its expression is closely connected with the expression of *CESA* genes in Arabidopsis (Li et al. 2003; Roudier et al. 2005). *OsPAL* was reported to regulate lignin synthesis in the phenylpropanoid pathway (Vanholme et al. 2008).

**Fig. 6:**
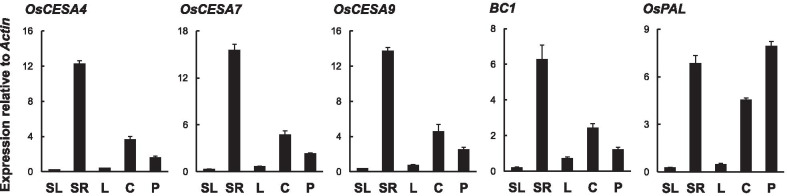
Expression analysis of *OsCESAs*, *BC1* and *OsPAL* relative to *Actin1* in different tissues of the wild type plant. Total RNA was isolated from seedling leaves (SL), seedling roots (SR), leaves and culms from plants two weeks after heading (L and C), young panicles (P). The rice *Actin1* was used as a control. Values represent averages of three independent replicates. Vertical bars show standard errors

As shown in Fig. 6, three *CESA* genes, *BC1* and *OsPAL* were mainly expressed in roots of seedlings, culms of plants two weeks after heading and young panicles at the beginning of heading stage. However, very low transcript levels of the five genes were detected in leaves at seedling stage and after heading. The only difference was that *OsPAL* was expressed much more strongly in young panicles than in culms after heading. Similar expression patterns of *CESAs* and *BC1* in different tissues suggested that they might be co-expressed in SCW synthesis, which was consistent with the results in the study of an *OsCESA9* mutant (Kotake et al. 2011). However, they only verified the relationship between *OsCESA9* and *BC1*. Additionally, we here confirmed the co-expression pattern between three *CESA* genes and *BC1*.

To seek if there was any effect on these related genes' expression caused by the mutation in *Bc19* gene, we then examined and compared them between *Bc19* mutant and the wild type. In *Bc19* mutant, expressions of the five genes were down-regulated in leaves and roots, while up-regulated in culms and panicles (Additional file 2: Fig. S4). However, changes in transcription levels of these genes were less than two folds in *Bc19* mutant, indicating that the point mutation in *Bc19* gene didn't necessarily affect *Bc19* or other related genes. A similar case occurred in the *bc1* mutant, where the expression levels of the three *CESA* genes were not affected by the mutation of gene *BC1* (Liu et al. 2013).

In addition, we compared transcript levels of *OsCESA4* among the homozygous mutant *Bc19*, the hybrid F_1_ plant, the wild type and two particularly brittle transgenic lines TG1 and TG2. As a result, the expression levels in *Bc19* mutant and F_1_ plants (the according transcripts probably including both the wild type and mutant gene, *BC19* and *Bc19*) were comparative to those in the wild type. However, sharply raised transcript levels of the *Bc19* gene were detected in TG1 and TG2 (Fig. 5d), showing inverse correlation with sharply decreased contents of cellulose, hemicellulose and lignin in them (Fig. 5b, c).

## Discussion

### *Bc19* Alters Mechanical Strength and Cell Wall Composition but Has No Distinct Influence on Growth and Grain Yield

**Fig. 7:**
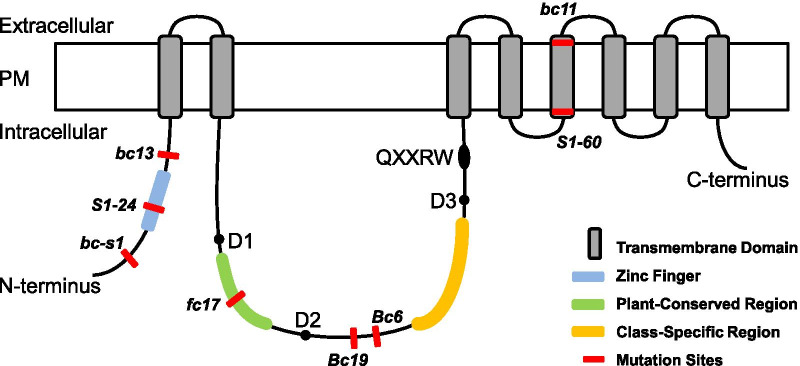
The predicted structure of a CESA protein showing the relative locations of reported point mutations of CESAs in rice. The basic domain structure of CESA was adapted from a review on cellulose synthesis in Arabidopsis (Li et al. 2014b). The open box stands for the plasma membrane (PM). A Zinc-finger domain (a blue bar) exists towards the N-terminus in the first cytoplasmic domain, followed by two transmembrane domains (TMDs; gray bars), the second cytoplasmic domain between the second and the third TMDs, and the other six TMDs. The Plant-conserved Region (P-CR, a green bar), the conserved D,D,D,QXXRW residues (black spots), and the Class-specific region (C-SR, a yellow bar) are all located in the second cytoplasmic domain. Red bars indicate point missense mutations as follows: *bc*-*s1* (OsCESA9, G10R, Jin et al. 2016), *S1*-*24* (OsCESA7, C40Y, Wang et al. 2016a), *bc13* (OsCESA9, G101K, Song et al. 2013), *fc17* (OsCESA4, F426S, Li et al. 2018), *Bc19* (OsCESA4, P507S) in this study, *Bc6* (OsCESA9, R588G, Kotake et al. 2011), *S1-60* (OsCESA9, G905D, Wang et al. 2012), and *bc11* (OsCESA4, G858R, Zhang et al. 2009)

Gene mutation of most brittle mutants affected mechanical strength of plant bodies and resulted in other pleiotropic abnormalities in rice, such as *bc3*, *bc10*, *bc12* (Hirano et al. 2010; Zhou et al. 2009; Zhang et al. 2010). Similarly, different mutation types and sites of CESAs also resulted in varied phenotypes in rice. NE1031, NC0259, ND8759, and ND2395 were generated by *Tos17* insertion in *OsCESA* genes, and they were aberrant in plant height, leaf size, culm thickness, and fertility (Tanaka et al. 2003). *bc7* was another brittle mutant allele of *OsCESA4* obtained through 60Co-γ radiation, resulting in the premature transcription of the corresponding gene (Yan et al. 2007). Plant height of *bc7* was shorter, and its cellulose content and cell number of the parenchymatous tissues were reduced. Other 7 *OsCESA* mutants were caused by point mutations as reported, including 2 of *OsCESA4* (*bc11* and *fc17*), 1 of *OsCESA7* (*S1*-*24*), and 4 of *OsCESA9* (*Bc6*, *S1*-*60*, *bc13*, and *bc*-*s1*) (Fig. 7) (Zhang et al. 2009; Li et al. 2018; Wang et al. 2016a, 2012; Kotake et al. 2011; Song et al. 2013; Jin et al. 2016). Among them, 3 mutants (*bc11*, *S1*-*24*, and *S1*-*60*) also showed shorter plant bodies, and/or other abnormal morphologies, such as poor fertility, fewer tillers and shorter roots. Most of those mutants had reduced cellulose contents and increased hemicellulose and lignin contents (Yan et al. 2007; Wang et al. 2016a, 2012; Kotake et al. 2011; Li et al. 2018). Here, we reported another brittle mutant *Bc19*, in which cellulose, hemicellulose and lignin contents were reduced by 12.5%—28.5%, but apparent morphology, growth and grain yield were not affected (Figs. 1, 3). Unlike most recessive brittle mutants such as *fc17*, we verified *Bc19* as a new semi-dominant mutant allele of gene *OsCESA4*, and the mutant gene mainly influenced the SCW synthesis (Figs. 1, 2, 3, 4).

In recent years, grain yield constantly attracts breeders' increasing concern about lodging resistance in rice cultivars. Enhancing culm strength can improve the lodging resistance in rice and confrontation capacity against natural disasters such as strong wind and rain (Li et al. 2014a). On the other hand, a reduced proportion of components in cell walls could make rice straw easily degraded after harvest, improving its utilization efficiency either as animal feed or biofuels (Wang et al. 2005; Johnson et al. 2006). In addition, engineering improvement of rice straw as biofuel resources will be a sustainable solution to avoid environmental problems caused by straw burning (Himmel et al. 2007; Ragauskas et al. 2014). In this study, cellulose content was reduced while growth and yield were not affected in *Bc19* mutant. Thus the mutant gene could be a potential genetic resource for rice straw recycling, which could also maintain grain yield (Figs. 1, 3). What's more, the specific semi-dominant characteristic of brittle gene *Bc19* makes it more convenient to implement high grain yield, heavy biomass, and efficient utilization in breeding grain-straw dual-purpose hybrid rice (Peng et al. 2010; Li et al. 2019).

### Possible Explanation for the Dominant-Negative Effect of the OsCESA4^P507S^ Mutation in ***Bc19*** Mutant

The P507S missense mutation, resulting in a semi-dominant effect in the *Bc19* mutant, was found located in the second cytoplasmic domain between TMD2 and TMD3 of OsCESA4. It's worth noting that this residue is corresponds to the P557T point mutation of AtCESA7 in the semi-dominant mutant *fra5* in Arabidopsis; and that the P507 residue was close to R509 which corresponds to the R588G mutation of OsCESA9 in the semi-dominant mutant *Bc6* in rice (Additional file 2: Fig. S2; Fig. 7) (Zhong et al. 2003; Kotake et al. 2011). This second cytoplasmic domain, providing glycosyltransferase activity for CESAs, consists of a Plant-Conserved Region (P-CR), a Class-Specific Region (C-SR), and a D,D,D,QXXRW motif (Fig. 7) (Somerville 2006; Li et al. 2014b). Available crystal structures of CESAs uncovered the hexamer of trimers model of CESA complex (CSC), which is formed by the aid of N-terminal stalk, interactions of the C-terminal TMD and trimerization of the cytosolic P-CRs with neighboring CESA subunits (Nixon et al. 2016; Purushotham et al. 2020; Qiao et al. 2021; Vandavasi et al. 2016). Although located in the cytosolic domain, these three semi-dominant mutation sites (OsCESA4^P507S^, OsCESA9^R588G^ and AtCESA7^P557T^) are situated between the P-CR domain and the C-SR domain, and distinct from the D,D,D,QXXRW motif (Fig. 7). It is possible that the highly conserved region between P-CR and C-SR has a certain function for glycosyltransferase activity other than formation of CSC. In this study, overexpression of *Bc19* gene in the wild type caused a more severe brittle phenotype than the *Bc19* mutant, while the mutant phenotype of F_1_ plants was milder than *Bc19* mutant (Figs. 5b-d, 3, 1a, b, j, k). It seems that the severity of the mutant phenotype is tightly related to the level of *Bc19* expression, implying that despite the dominant-negative effect, *Bc19* gene might also act in a dosage way. Moreover, the predicted 3D structure of CESA4^P507S^ was perfectly overlapped with the wild type CESA4 (Additional file 2: Fig. S3). Considering these facts, we presume that the mutated version of OsCESA4, CESA4^P507S^, has a defect in glycosyltransferase activity but has no effect on CSC formation, thus it can interact with other normal CESA subunits and composes into the CSC complex, resulting in the dominant-negative effect of a dose-dependent fashion. On the other hand, point mutations located in Zinc Finger domain, P-CR domain or TMDs make the corresponding mutated CESA subunits fail to interact with normal subunits, causing the recessive phenotypes of those brittle mutants, such as *S1*-*24*, *fc17* and *bc11* (Fig. 7) (Wang et al. 2016a; Li et al. 2018; Zhang et al. 2009). Intensive exploration of the action mechanism of P-CR of CESA subunits and activities between components within the CSC complex will help to uncover how these P-CRs participate in cellulose biosynthesis.

## Conclusions

*Bc19*, a semi-dominant brittle mutant allele of gene *OsCESA4*, was identified using a map-based cloning approach. Breaking force and cellulose content of the *Bc19* mutant were decreased, while its apparent morphology, growth and grain yield were not affected. The product encoded by *Bc19* possessed a P507S missense mutation located in the second cytoplasmic region, causing a dominant-negative effect. The relationship between *Bc19* expression and brittleness severity further revealed that gene *Bc19* might affect cellulose synthesis in a dosage-dependent manner. Moreover, we presumed that the *Bc19* gene could be a potential genetic resource in the breeding of grain-straw dual-purpose hybrid rice.

## Materials and Methods

### Plant Materials and Growth Conditions

The *Brittle culm19* (*Bc19*) mutant was obtained from the *japonica* cv. Nipponbare through ethyl methanesulfonate (EMS) mutagenesis. Then Nipponbare, the *indica* cv. Minghui 63 and G46B were crossed with *Bc19* to obtain three F_1_ hybrids, respectively. Due to the better polymorphism between Nipponbare and Minghui 63, the F_2_ mapping population was generated from selfing F_1_ plants of *Bc19* and Minghui 63. Rice plants were cultivated in the local fields in Wenjiang District (Latitude 30˚42′N, Longitude 103˚50′E, and Altitude 539.3 m), Chengdu City, Sichuan, China (Wang et al. 2010).

### Measurement of Major Agronomic Traits

*Bc19*, Nipponbare, and transgenic plants were cultivated according to a randomized complete block design with three replications. Each block contained 40 plants, and the planting density was 16.6 × 25 cm. Field management followed local rice production. Breaking force of flag leaves and the top second internodes were measured two weeks after heading with a digital force testing device (FGJ-1, SHIMPO). The force to break apart a leaf or culm segment was recorded accordingly. Other agronomic traits and yield per plant were investigated after maturation. All of the data were calculated using the software IBM SPSS Statistics 22, and the statistical significance of differences between *Bc19* and the wild type plant was conducted using Student's t-test.

### Analysis of Cell Wall Components

Two weeks after heading, flag leaves and the top second internodes were dried at 105 ℃ for 1 h and then at 65 ℃ for 24 h. Afterward, the materials were ground into fine powder. Contents of cellulose, hemicellulose and lignin were measured according to the methods previously described by Van Soest et al. (1991). For measurement of cell wall sugars, the powdered materials were soaked in 80% acetonitrile, and incubated in a 65 ℃ ultrasonic oscillation water bath for 30 min. The supernatants were collected through a centrifuge, followed by the analyzing monosaccharides with HPLC system (Agilent 1260A) according to the methods reported (Zhao et al. 2020).

### Electron Microscopy Analysis

An environmental scanning electron microscope (ESEM, FEI-Q450) was used to observe structure of sclerenchyma and parenchyma tissues. Flag leaves and the top second internodes were cut into small pieces and then immediately placed on the object stage for observation.

For transmission electron microscope analysis, sections of leaf tissues at seedling stage were treated as previously described (Wang et al. 2010). Generally, these sections were fixed in 3% (w/v) glutaraldehyde overnight and then in 1% osmium tetroxide. After that, the samples went through a gradient of ethanol series to get dehydrated, followed by washing with the Epon812 medium. Then, the samples were cut into ultra-thin sections and stained with uranyl acetate and Reynolds' lead citrate. Finally, the slices were observed using the transmission electron microscope (H-600IV, Hitachi).

### Fine Mapping and Marker Development

Nine hundred and eighty-three normal plants selected from an F_2_ population up to 5000 plants from the cross between *Bc19* and Minghui 63 were used for gene mapping. Genetic linkage analysis was conducted using simple sequence repeat (SSR) markers (McCouch et al. 2002), taking genomic DNA as templates. Two DNA pools (normal/brittle), each mixed with 10 individuals, were constructed to screen those SSR markers. Markers showing different bands between the two pools would be further confirmed by the F_2_ segregation population. Insertion/deletion (InDel) markers were designed using Primer Premier 5.0 software based on genome sequence polymorphism between *japonica* and *indica* from the NCBI website (http://www.ncbi.nlm.nih.gov/BLAST).

### Vector Construction and Transgenic Experiment

The cDNA sequence of the mutant *Bc19* gene was amplified from the mutant with primers 5′-GGGTCTAGAATGATGGAGTCGGGGGTC-3′ and 5′-AAAGTCGACTCAGCAGTCGAAGTTGGC-3′ (containing a *Xba*I site and a *Sal*I site, respectively), and then inserted into the pMD19-T vector (TaKaRa). After sequencing, the plasmid was double digested with enzymes *Xba*I and *Sal*I. Subsequently the resulting *Bc19* fragment was inserted into the binary vector pCAMBIA2300 behind the rice *Actin1* promoter. The construct *pC2300*-*Bc19* was introduced into the wild type plant Nipponbare by *Agrobacterium tumefaciens*-mediated transformation (Kumar et al. 2005). Primers for identifying the transgenic plants were 5′-GAATCCCTCAGCATTGTTC-3′ and 5′-TCAAATGTGAGCATAGCC-3′, with annealing sites on the rice *Actin1* promoter and cDNA of *Bc19*, respectively. We chose two homozygous lines (TG1 and TG2) from eleven positive transgenic lines for the test of cell wall components and qRT-PCR.

### Protein Structure Prediction and Sequence Alignment

Amino acid sequences of CESAs in rice and Arabidopsis were downloaded from GenBank (http://www.ncbi.nlm.nih.gov) through the BLAST program. Transmembrane domains of CESAs were predicted using the TMHMM program (http://www.cbs.dtu.dk/services/TMHMM). Multiple sequence alignments were conducted using DNAMAN software (Lynnon Biosoft). Three dimensional (3D) structures of OsCESA4 and Bc19 (OsCESA4^P507S^) were predicted by alphafold2, and the alignment of them was performed using pymol software (https://www.deepmind.com/research/case-studies/alphafold; https://pymol.org/2/).

### qRT-PCR Analysis

Total RNA from leaves and roots of two-week-old seedlings, from flag leaves and top second internodes of plants two weeks after heading, and from young panicles at the beginning of heading stage was extracted with a TRIzol reagent (Invitrogen). The first-strand cDNA was produced using a Superscript III Reverse Transcription Kit (Invitrogen). We designed specific primers for each gene, and chose rice *Actin1* gene as an internal control (Additional file 1: Table S3). Quantitative RT-PCR (qRT-PCR) was performed using an SYBR Premix Ex Taq2 kit (TaKaRa) according to the following program: 95 °C for 5 min, then 40 cycles of 95 °C for 10 s and 58 °C for 30 s. For each experimental group, qRT-PCR was operated with three technical replicates for each of the three biological replicates.

## Supplementary Information

**Additional file 1: Table S1.** Segregation in the F_2_ population from crossing of *Bc19* with normal cultivars. **Table S2.** Insertion/deletion (InDel) markers developed for fine mapping of the *Bc19* locus. **Table S3.** Primers used in qRT-PCR.

**Additional file 2: Fig. S1.** Part of alignments of amino acid sequences of OsCESA4 with similar sequences in other CESA family members of rice and Arabidopsis (**a**) and in different species (**b**). **Fig. S2.** Alignment of amino acid sequences of three main kinds of CESAs in rice and Arabidopsis. **Fig. S3.** Predicted three dimensional (3D) structures of OsCESA4 and Bc19 (OsCESA4^P507S^) proteins. **Fig. S4.** Comparison of *OsCESAs*, *BC1* and *OsPAL* mRNAs between WT and *Bc19* mutant.

## Data Availability

All data generated or analyzed during this study are included in this article (and its supplementary information files).

## Abbreviations

CESA: Cellulose synthase A
PCW: Primary cell walls
SCW: Secondary cell walls
P-CR: Plant-conserved region
C-SR: Class-specific region
TMD: Transmembrane domains
CSC: Cellulose synthase complex
