# Supplementary material for: Rice Brittle Culm19 Encoding Cellulose Synthase Subunit CESA4 Causes Dominant Brittle Phenotype But has No Distinct Influence on Growth and Grain Yield
Source: Rice (N Y). 2021 Nov 25;14:95. doi: 10.1186/s12284-021-00536-2 (PMC8617145; doi:10.1186/s12284-021-00536-2)
Supplement: Supplementary file 1 — Additional file 1: Table S1. Segregation in the F2 population from crossing of Bc19 with normal cultivars. Table S2. Insertion/deletion (InDel) markers developed for fine mapping of the Bc19 locus. Table S3. Primers used in qRT-PCR. [file 12284_2021_536_MOESM1_ESM.doc]

**Supplemental Table S1** Segregation in the F_2_ population from crossing of *Bc19* with normal cultivars.

| Cross-combination | No. of Total plants | No. of brittle plants | No. of normal plants | Expected ratio | χ^2^ |
| --- | --- | --- | --- | --- | --- |
| Nipponbare/*Bc19* | 216 | 166 | 50 | 3:1 | 0.30 |
| Minghui 63/*Bc19* | 203 | 158 | 45 | 3:1 | 0.72 |
| G46B/*Bc19* | 219 | 171 | 48 | 3:1 | 0.95 |

**Supplemental Table S2 Insertion/deletion (InDel) markers developed for fine mapping of the *Bc19* locus.**

| Maker | Forward primer (5′*–*3′) | Reverse primer (5′*–*3′) |
| --- | --- | --- |
| C1 | TACTAGATATGCCTCTTTCG | TGGATAATAAACTATGGTGG |
| C2 | ACCAAATCAGGGAAACAA | GGTCAAGACGGTCAAGGA |
| C3 | GTGTATAACCAACCGAAC | ATCTGTCTCACGGATGAC |
| C4 | ATGGACGGCTCATTGTTG | CTTGCGATGGCATTTGTT |
| C5 | TGGAGACTTCGAGGATTG | GCTGCTGTGAACACATAG |
| C6 | TCCAGGAAGGCAGACATT | TAGCAGTTTGCAGGGTTG |

**Supplemental Table S3** Primers used in qRT-PCR.

| Gene | Forward primer (5'-3') | Reverse primer (5'-3') |
| --- | --- | --- |
| *Actin1* | TGTATGCCAGTGGTCGTACCA | CCAGCAAGGTCGAGACGAA |
| *CESA4* | CTCCGAGACCACCACCACCAAC | ACCCATCGTCTTCGTCGCATTAG |
| *CESA7* | AAGCCATGCGGGGTCTCGTG | CATCCATCCGGTCATCCCTCTTG |
| *CESA9* | ATCGCGCTCTTCATCTCCATCTTC | ACTGCTCGTTCCTCCACCACTCC |
| *BC1* | CTTTGAAATTGCCTGATAGA | AAAGTTTGTGGTGTGATTT |
| *OsPAL* | ACCGCTTCGTGTATCTTCAG | AAGGATGGAATCGAGTAGCA |
