# Supplementary material for: Rice Brittle Culm19 Encoding Cellulose Synthase Subunit CESA4 Causes Dominant Brittle Phenotype But has No Distinct Influence on Growth and Grain Yield
Source: Rice (N Y). 2021 Nov 25;14:95. doi: 10.1186/s12284-021-00536-2 (PMC8617145; doi:10.1186/s12284-021-00536-2)
Supplement: Supplementary file 2 — Additional file 2: Fig. S1. Part of alignments of amino acid sequences of OsCESA4 with similar sequences in other CESA family members of rice and Arabidopsis (a) and in different species (b). Fig. S2. Alignment of amino acid sequences of three main kinds of CESAs in rice and Arabidopsis. Fig. S3. Predicted three dimensional (3D) structures of OsCESA4 and Bc19 (OsCESA4P507S) proteins. Fig. S4. Comparison of OsCESAs, BC1 and OsPAL mRNAs between WT and Bc19 mutant. [file 12284_2021_536_MOESM2_ESM.pdf]

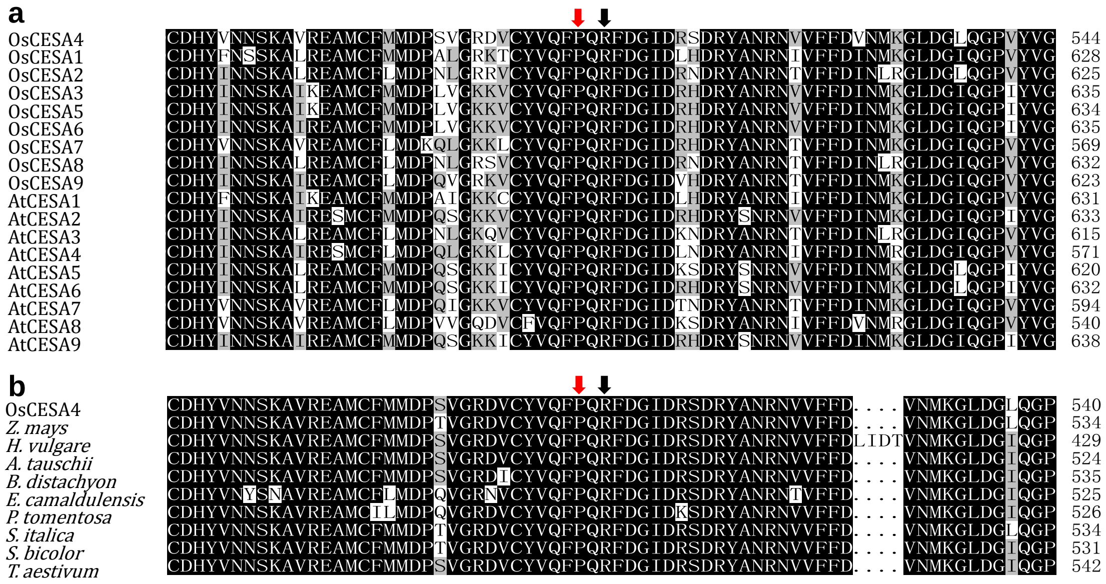

Supplementary Figure S1 Part of alignments of amino acid sequences of OsCESA4 with similar sequences in other CESA family members of rice and Arabidopsis (**a**) and in different species (**b**). Dark and gray backgrounds showed the identical and similar residues, respectively. A red arrow indicates the missense mutation P507S in mutant *Bc19*, which is strictly conserved and is only one residue away from the R588G mutation in mutant *Bc6* (black arrow, Kotake et al. 2011). Accession numbers for these sequences are as follows: *Oryza sativa* OsCESA4 (LOC_Os01g54620, Q5JN63.1), OsCESA1 (LOC_Os05g08370, Q6AT26.1), OsCESA2 (LOC_Os03g59340, Q84M43.1), OsCESA3 (LOC_Os07g24190, Q69V23.1), OsCESA5 (LOC_Os03g62090, Q851L8.1), OsCESA6 (LOC_Os03g62090, Q6YVM4.1), OsCESA7 (LOC_Os10g32980, Q9AV71.1), OsCESA8 (LOC_Os07g10770, Q84ZN6.1), and OsCESA9 (LOC_Os09g25490, Q69P51.1); *Arabidopsis thaliana* AtCESA1 (At4g32410, NP_194967.1), AtCESA2 (At4g39350, NP_195645.1), AtCESA3 (At5g05170, NP_196136.1), AtCESA4 (At5g44030, Q84JA6.1), AtCESA5 (At5g09870, Q8L778.2), AtCESA6 (At5g64740, Q94JQ6.2), AtCESA7 (At5g17420, NP_197244.1), AtCESA8 (At4g18780, NP_567564.1), and AtCESA9 (At2g21770, Q9SJ22.1); *Zea mays* (DAA36974.1); *Hordeum vulgare* (AAR29965.1); *Aegilops tauschii* (EMT23140.1); *Brachypodium distachyon* (XP_003569818.1); *Eucalyptus camaldulensis* (AEK31215.1); *Populus tomentosa* (AEE60894.1); *Setaria italica* (XP_004969957.1); *Sorghum bicolor* (XP_002456361.1); and *Triticum aestivum* (CBH32503.1).

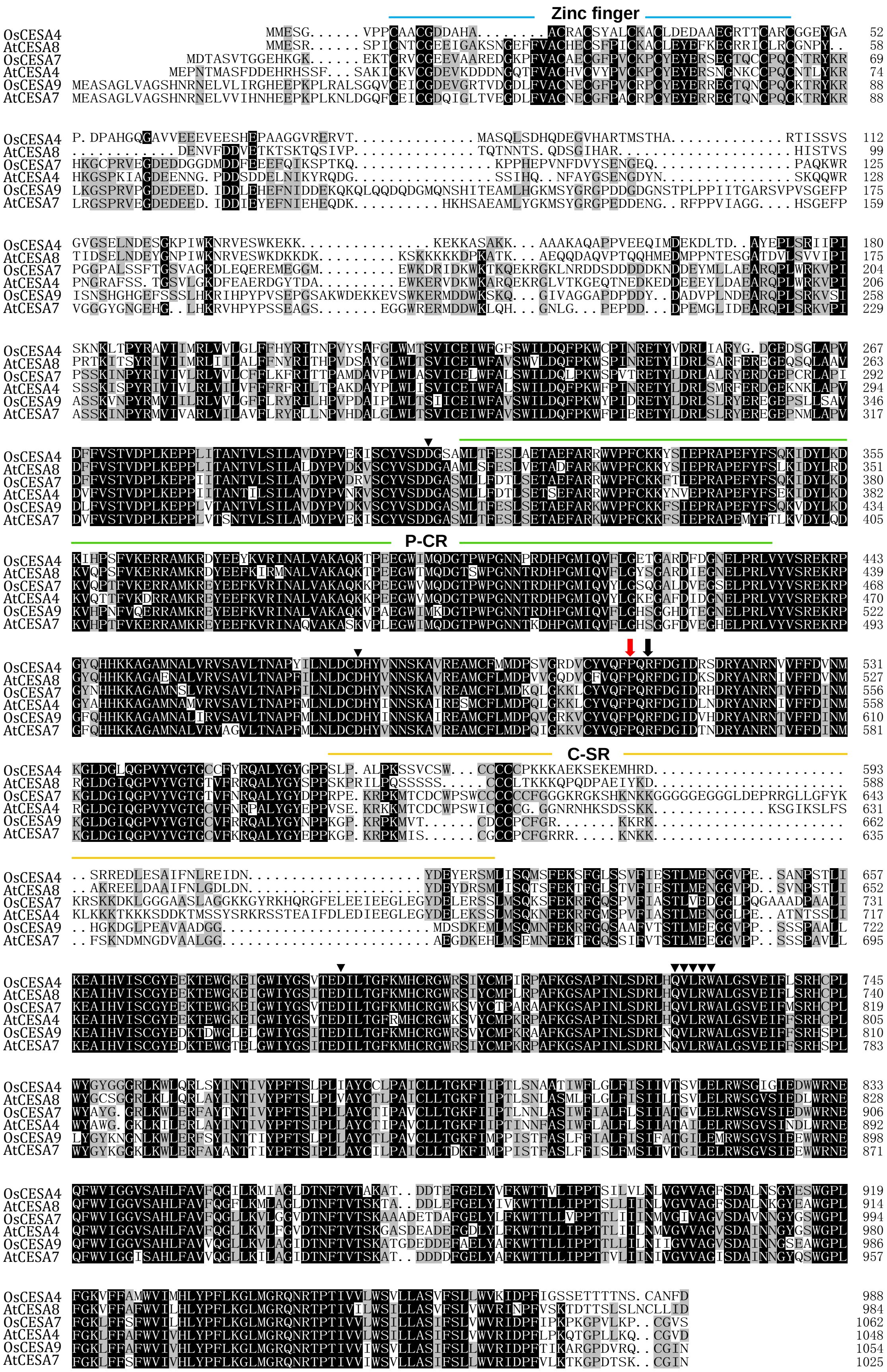
Zinc finger
P-CR
C-SR
OsCESA4
AtCESA8
OsCESA7
AtCESA4
OsCESA9
AtCESA7

Supplementary Figure S2 Alignment of amino acid sequences of three main kinds of CESAs in rice and Arabidopsis. Dark and gray backgrounds showed the identical and similar residues, respectively. The Zink finger domain, Plant-Conserved Region (P-CR) and Class-Specific Region (C-SR) are overlined in blue, green, and yellow, respectively, and inverted triangles point out the D,D,D,QXXRW motif. A red arrow indicates the missense mutation P507S in mutant *Bc19*, which is strictly conserved and is only one residue away from the R588G mutation in mutant *Bc6* (black arrow, Kotake et al. 2011). GenBank accession numbers for these sequences are as Supplementary Fig. S1.

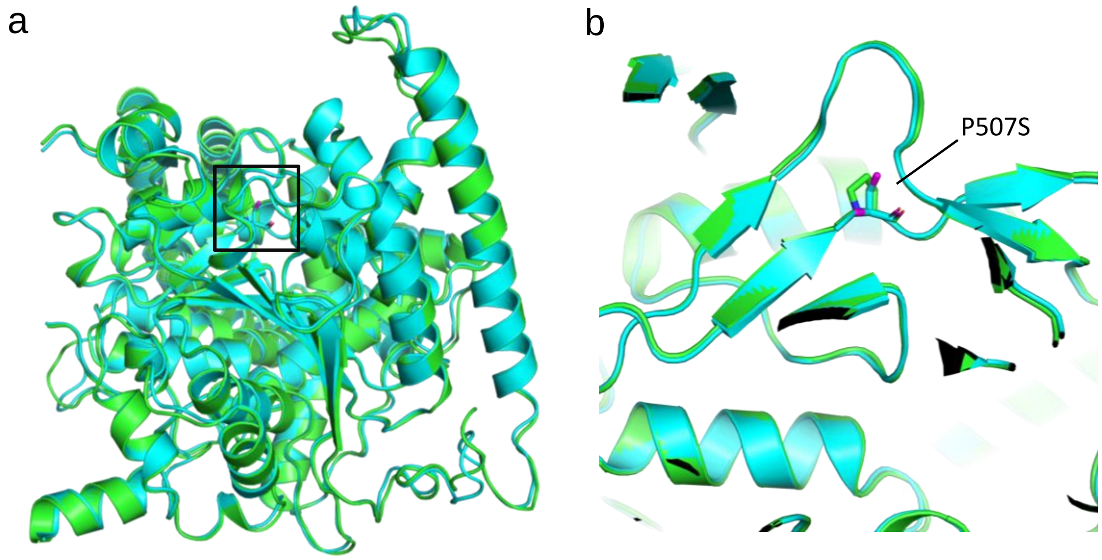

Supplementary Figure S3 Predicted three dimensional (3D) structures of OsCESA4 and Bc19 (OsCESA4^{P507S}) proteins. **a** Alignment of the wild type OsCESA4 (green) and the P507S mutated Bc19 (OsCESA4^{P507S}, blue). **b** The magnified section of **a**, showing the P507S mutation between two β-pleated sheets (thick arrow).

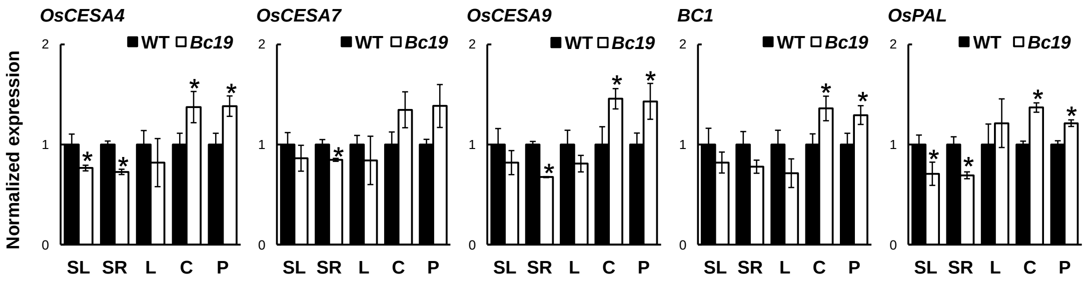

Supplementary Figure S4 Comparison of *OsCESAs*, *BC1* and *OsPAL* mRNAs between WT and *Bc19* mutant. Total RNA was isolated from seedling leaves (SL), seedling roots (SR), leaves and culms from plants two weeks after heading (L and C), young panicles (P). The rice *Actin1* was used as a control. Values represent averages of three independent replicates. Vertical bars show standard errors. The expression data of WT were all set to 1.0 and those of *Bc19* were adjusted accordingly. Asterisks signify statistically significant differences compared to WT at $P < 0.05$.
